# Discovery of Novel Lipid Profiles in PCOS: Do Insulin and Androgen Oppositely Regulate Bioactive Lipid Production?

**DOI:** 10.1210/jc.2016-2692

**Published:** 2016-11-28

**Authors:** Shengxian Li, Qianqian Chu, Jing Ma, Yun Sun, Tao Tao, Rong Huang, Yu Liao, Jiang Yue, Jun Zheng, Lihua Wang, Xinli Xue, Mingjiang Zhu, Xiaonan Kang, Huiyong Yin, Wei Liu

**Affiliations:** 1 Department of Endocrinology and; 2 Shanghai Key Laboratory for Assisted Reproduction and Reproductive Genetics, Center for Reproductive Medicine and; 3 Department of Biobank, Renji Hospital, School of Medicine, Shanghai Jiao Tong University, Shanghai 200127, China;; 4 Key Laboratory of Food Safety Research and; 5 Mass Spectrometry Research Center, Institute for Nutritional Sciences, Shanghai Institutes for Biological Sciences, Chinese Academy of Sciences, Shanghai 200031, China;; 6 Key Laboratory of Food Safety Risk Assessment, Ministry of Health, Beijing 100021, China;; 7 University of the Chinese Academy of Sciences, Shanghai 200031, China; and; 8 School of Life Science and Technology, ShanghaiTech University, Shanghai 200031, China

## Abstract

**Context:**

Polycystic ovary syndrome (PCOS) is a complex syndrome showing clinical features of an endocrine/metabolic disorder, including hyperinsulinemia and hyperandrogenism. Polyunsaturated fatty acids (PUFAs) and their derivatives, both tightly linked to PCOS and obesity, play important roles in inflammation and reproduction.

**Objective:**

This study aimed to investigate serum lipid profiles in newly diagnosed patients with PCOS using lipidomics and correlate these features with the hyperinsulinemia and hyperandrogenism associated with PCOS and obesity.

**Design and Setting:**

Thirty-two newly diagnosed women with PCOS and 34 controls were divided into obese and lean subgroups. A PCOS rat model was used to validate results of the human studies.

**Main Outcome Measures:**

Serum lipid profiles, including phospholipids, free fatty acids (FFAs), and bioactive lipids, were analyzed using gas chromatography–mass spectrometry (MS) and liquid chromatography–MS.

**Results:**

Elevation in phosphatidylcholine and a concomitant decrease in lysophospholipid were found in obese patients with PCOS vs lean controls. Obese patients with PCOS had decreased PUFA levels and increased levels of long-chain saturated fatty acids vs lean controls. Serum bioactive lipids downstream of arachidonic acid were increased in obese controls, but reduced in both obese and lean patients with PCOS vs their respective controls.

**Conclusions:**

Patients with PCOS showed abnormal levels of phosphatidylcholine, FFAs, and PUFA metabolites. Circulating insulin and androgens may have opposing effects on lipid profiles in patients with PCOS, particularly on the bioactive lipid metabolites derived from PUFAs. These clinical observations warrant further studies of the molecular mechanisms and clinical implications of PCOS and obesity.

## Teaser

We found serum lipid profiles changed in women with PCOS. Obesity and compensatory hyperinsulinemia promoted the metabolism of arachidonic acid and other PUFAs, whereas androgen had an inhibitory effect.

Polycystic ovary syndrome (PCOS) is a complex multisystem syndrome that occurs in postpubertal women. According to diagnostic criteria of the National Institute of Child Health and Human Development/National Institutes of Health, 4% to 10% of women of reproductive age have PCOS (1–3). The major manifestations of PCOS are dilute ovulation/anovulation, polycystic ovaries, and hyperandrogenism. PCOS is often associated with obesity, insulin resistance, abnormal glucose tolerance, lipid metabolic disorders, and other metabolic abnormalities (4, 5). The prevalence of obesity in women with PCOS is approximately 50% to 80% (4). Obesity is associated with abnormal adipokine secretion, elevated serum free fatty acids (FFAs), and metabolic disorders associated with abnormal steroid hormone function in adipose tissue, resulting in low-grade chronic inflammation, insulin resistance, and abnormal glucose tolerance (4, 6). Approximately 20% of obese patients with PCOS have impaired glucose tolerance or type 2 diabetes mellitus (T2DM) (7); in addition, lean patients with PCOS show increased prevalence of impaired glucose tolerance and T2DM compared with women without PCOS (5). The prevalence of insulin resistance is between 44% and 70% (4, 8, 9), and it manifests as impaired insulin-mediated suppression of lipolysis and lipid oxidation (10, 11), resulting in increased serum FFAs in obese women with PCOS that are matched for body mass index (BMI) (12).

Another core pathophysiologic feature of PCOS is hyperandrogenism. Androgen promotes lipolysis in visceral fat cells, which is an early and possibly primary metabolic defect in PCOS (13, 14). However, how insulin and hyperandrogenism contribute to the adverse lipid profile present in PCOS, which includes altered concentrations of phospholipids, FFAs, and bioactive lipid metabolites derived from polyunsaturated fatty acids (PUFAs), remains poorly defined. Notably, prostaglandins (PGs), which are cyclooxygenase (COX)–generated metabolites of arachidonic acid (AA), influence both the development of reproductive defects and chronic inflammation, which are hallmarks of PCOS. As a result of defects in follicular maturation, COX knockout mice show abnormal ovulation and fertilization of their oocytes (15–17). COX inhibition can also block blastocyst development, which suggests that COX and its products are involved in early embryonic development (18). In addition, PGE_2_, PGI_2_, PGF_2_*_α_*, PGD_2_, and 15-deoxy-Δ^12,14^-PG J_2_ also play major roles in follicular/oocyte development, ovulation, and fertilization (19–23). Furthermore, PGs are involved in the chronic inflammation that develops alongside the metabolic disorders associated with PCOS and obesity. However, the circulating levels of these important lipid mediators have not been systematically defined in patients with PCOS, and it is unknown how obesity and hyperandrogenism affect the production of these bioactive lipids in these patients.

**Figure 1.**
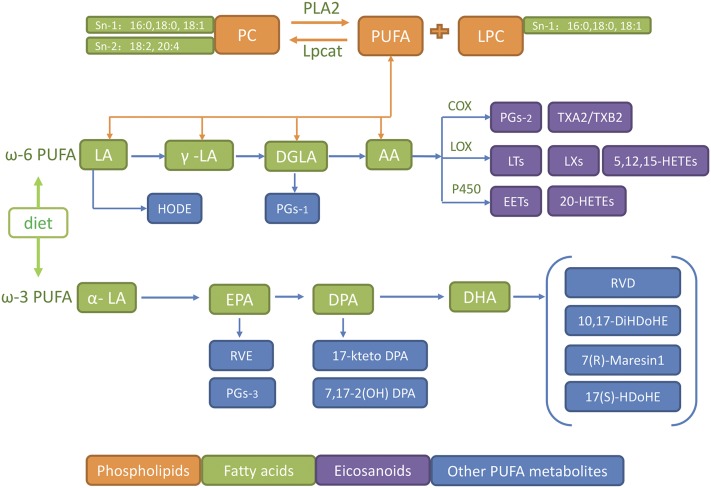
Pathways whereby bioactive lipid mediators are generated from phospholipids and FAs. AA is C20:4; DHA is C22:6; LA is C18:2. ALA, alpha linolenic acid C18:3*α*; DGLA, dihomo-*γ*-linolenic acid C18:3*γ*; DPA, docosapentaenoic acid C22:5; EPA, eicosapentaenoic acid C20:5; Lpcat, lysophosphatidylcholine acyltransferase; LT, leukotriene; LX, lipoxin; PLA_2_, phospholipase A2; P450epo, cytochrome P450 epoxygenase; RVD, resolvin D; RVE, resolvin E; TXA2, thromboxane A2.

As a branch of metabolomics, lipidomics is used to systematically investigate a large range of lipids in a given biological system. It has become an indispensable tool to study lipid metabolism in human disease. In this study, we carried out systematic lipidomic profiling of the serum lipids that comprise the major lipid metabolic pathways (Fig. 1) in lean and obese patients with PCOS and matched control subjects using mass spectrometry (MS)–based lipidomic methods. We observed significantly higher levels of phosphatidylcholine (PC) and concomitantly lower lysophospholipid (LPC) in obese PCOS groups. Concentrations of PUFAs, such as AA, linoleic acid (LA), and docosahexaenoic acid (DHA), were also lower, whereas saturated long-chain fatty acids (FAs) were higher. Interestingly, however, major downstream bioactive lipids generated from PUFAs were significantly higher in sera from obese controls and lower in obese and lean patients with PCOS when compared with their respective controls. These data suggest that obesity, which is typically accompanied by hyperinsulinemia, stimulates the production of bioactive lipids, whereas androgen has the opposite effect. Similar results were obtained using a rat model of PCOS that is obese and demonstrates hyperandrogenism. Our clinical observations imply that further mechanistic studies should be undertaken with regard to the antagonistic effects of insulin and androgen on circulating lipids in patients with PCOS. A deeper understanding of the molecular mechanisms that are involved in the hormonal and metabolic disorders present in PCOS may be able to guide new clinical interventions in future.

## Materials and Methods

### Subjects

This clinical study was approved by the Internal Review and Ethics Boards of Renji Hospital, which is affiliated with Shanghai Jiao Tong University. All of the participants signed informed consent to be included in the study. Both newly diagnosed patients with PCOS and healthy controls were enrolled in the clinics of Renji Hospital. The study subjects were divided into 4 groups: lean control (LC; BMI <24 kg/m^2^; n = 18), obese (OB; BMI ≥28 kg/m^2^; n = 16), obese PCOS (OP; BMI ≥28 kg/m^2^; n = 15), and lean PCOS (LP; BMI <24 kg/m^2^; n = 17), on the basis of the Chinese criteria for BMI categories (24). The diagnostic criteria for PCOS were based on the unified standards formulated by the Rotterdam International Conference in 2003. Patients with any 2 of the following 3 conditions were diagnosed with PCOS: (1) infrequent ovulation or anovulation; (2) hyperandrogenism or clinical manifestations of high blood androgen; (3) ultrasound findings of polycystic ovaries in 1 or 2 ovaries, or ≥12 follicles measuring 2 to 9 mm in diameter, and/or ovarian volume ≥10 mL. Exclusion criteria included congenital adrenal hyperplasia, androgen-secreting tumors, Cushing syndrome, thyroid dysfunction, hyperprolactinemia, and other diseases (25). Patients in the control groups exhibited normal menstruation, no clinical or biochemical signs of hyperandrogenism, normal ovaries as defined by B-mode ultrasonic examination, no family history of endocrine and metabolic diseases, and no family history of PCOS.

### Clinical measurements

Fasting plasma glucose concentration was quantified using the glucose oxidase method. Serum lipids [total cholesterol, triglyceride (TG), high-density lipoprotein (HDL), and low-density lipoprotein (LDL)] were measured by enzymatic assays (Cobas auto analyzer; Roche Diagnostics, Basel, Switzerland). Serum insulin was measured by radioimmunoassay. The concentrations of serum hormones [follicle stimulating hormone, luteinizing hormone, sex hormone–binding globulin (SHBG), and dehydroepiandrosterone sulfate (DHEAS)] were determined using chemiluminescence (Elecsys Auto analyzer; Roche Diagnostics). Total testosterone (TT) was measured by liquid chromatography–MS according to protocols previously reported (26). The intra-assay and interassay coefficients of variation were <6% and 10%, respectively, for all analyses. The insulin resistance index was calculated as homeostatic model assessment–insulin resistance (HOMA-IR). HOMA-IR = (fasting plasma glucose × fasting serum insulin) / 22.5. The free androgen index (FAI) was calculated as (TT × 100) / SHBG. The subjects also received an ultrasound examination in the Department of Obstetrics and Gynecology on a GE Voluson E8 System (GE Healthcare, Madison, WI) between the second and fifth days after menstruation.

### Lipidomics/metabolomics

Solvents for sample preparation and MS analysis, such as methanol, chloroform, and water, were purchased from Burdick and Jackson (Muskegon, MI). Other high-performance liquid chromatography–quality solvents, including methanol, water, 2-propanol, hexane, and acetonitrile, were purchased from either Fisher Chemical (Phillipsburg, NJ) or EM Science (Gibbstown, NJ). Standards and deuterated standards for AA metabolites, including PGD_2_, PGE_2_, PGF_2_*_α_*, PGI_2_, thromboxane B2 (TXB_2_), hydroxyeicosatetraenoic acids (HETEs), hydroxyoctadecadienoic acids (HODEs), and epoxyeicosatrienoic acids (EETs), were purchased from Cayman Chemical (Ann Arbor, MI) and used without further purification. Fatty acids of the highest purity (>99%) were purchased from Nu-Chek Prep (Elysian, MN). All other chemical reagents were from Sigma-Aldrich (St. Louis, MO).

All lipid extracts from human or rat plasma were prepared as described previously, with slight modifications (27, 28). For plasma lipidomics, 200 μL of plasma was extracted after addition of the internal standard mixture. Shotgun lipidomics was performed on a TSQ Vantage triple-quadrupole mass spectrometer (Thermo Scientific, San Jose, CA) according to published protocols (28). Tandem MS scan fragment and collision energy for each lipid class were optimized as previously reported (27, 28). Fatty acids were analyzed as fatty acid methyl esters using gas chromatography (GC)–MS (Agilent 6890N/5975B; Agilent, Santa Clara, CA) in the positive-ion mode of electron impact–MS according to published protocols (29, 30). Fatty acid methyl esters were analyzed by GC (Agilent 6890 GC with SP-2560 capillary column; Agilent; and 100 m × 0.25 mm × 0.2 μm film; Supelco, Bellefonte, PA).

Fatty acid metabolites of AA and other PUFAs were analyzed using a previously reported targeted metabolomic method (31–33). Briefly, after addition of a mixture of deuterated internal standards to 400 μL of serum, the pH of the solution was adjusted to 3.0 using 1 N HCl. Liquid-liquid extraction of the mixture was carried out twice using hexane:methyl *t*-butyl ether (50:50, v/v). The samples were separated on a Phenomenex Kinetix C18 column (3 μm, 100 × 2.1 mm; Phenomenex, Torrance, CA) using a Thermo Accela UPLC system (Thermo Scientific) at a rate of 0.4 mL/min, using a gradient of mobile phase A (water:acetonitrile:formic acid 63:37:0.02, v/v/v) and mobile phase B (acetonitrile:isopropanol 50:50, v/v). MS analysis was carried out on a TSQ Vantage triple-quadrupole mass spectrometer (Thermo Scientific). The mass spectrometer was operated in the negative ion mode using multiple reaction monitoring. Data acquisition and analysis were performed using Xcalibur software, version 2.0 (Thermo Scientific).

### Animal experiments

Animal experiments were performed according to protocols approved by the Animal Care Committee of the School of Medicine affiliated with Shanghai Jiaotong University. Thirty 21-day-old female Sprague-Dawley rats (50 to 60 g) from the Shanghai Laboratory Animal Center of the Chinese Academy of Science, Shanghai, China, were individually housed in ventilated cages in a temperature- and humidity-controlled environment. The animals were randomly divided into 3 groups: normal controls (CON; n = 10), a high-fat-diet–fed group (HF; n = 10), and a high-fat-diet–fed and hyperandrogenism group (HF + DHEA; n = 10), the last being composed of obese rats with PCOS and high levels of androgen. Rats in the HF + DHEA group were fed a high-fat diet and subcutaneously injected with DHEA (Sigma-Aldrich) daily at a dose of 6 mg/100 g of body weight in 0.2 mL of oil as a vehicle for 8 weeks, whereas the control group was fed a normal diet and injected with vehicle only. The high-fat diet comprised lard (20%), sugar (4%), whole milk powder (2%), cholesterol (1.5%), cholate (0.75%), plus a basal diet component (71.75%, containing 20% protein, 4% fat, 5% crude fiber, 8% crude ash, and 52.5% nitrogen-free extract). When the body mass of the HF group became 30% greater than that of the control group and assessment of vaginal smears showed a disappearance of estrous cyclicity in the HF + DHEA group, fasting blood was collected for the measurement of biochemical parameters including glucose, insulin, lipids, and sex hormones, as well as for MS analysis for FFAs and lipid metabolites. Rat ovaries were also collected for paraffin embedding and hematoxylin and eosin staining to evaluate follicular changes. Vaginal smears were evaluated microscopically after Wright-Giemsa staining.

### Statistical analysis

All data were analyzed using SPSS, version 16.0 (SPSS, Chicago, IL). Continuous data are presented as means ± standard errors of the mean. The nonnormal distribution data were analyzed after logarithmic transformation. Intergroup comparisons were performed using 1-way analysis of variance, and categorical variables were analyzed using the *χ*^2^ test. The Mann-Whitney *U* test was used to analyze nonparametric data. *P* < 0.05 was considered statistically significant.

## Results

### Characteristics of the study subjects

The characteristics of the 4 groups of study subjects are summarized in Table 1. The average age of the LP group was lower than that of groups LC, OB, or OP. The OP group showed more marked metabolic disturbances than the other 3 groups, including higher levels of fasting blood glucose, fasting serum insulin, HOMA-IR, TG, and LDL, as well as lower levels of HDL. Higher levels of TT and DHEAS were observed in subjects with PCOS (OP and LP groups) vs those with no PCOS (LC and OB groups), whereas there were no significant differences within PCOS groups. The level of SHBG in the OP group was markedly lower than that in the LP group, whereas the FAI was significantly higher in the OP group than in the LP group. Interestingly, the incidence of nonalcoholic fatty liver disease was much higher in the PCOS groups (OP and LP) than in their respective non-PCOS control groups (OB and LC).

**Table 1.** **Characteristics of the Study Participants**

	LC (n = 18)	OB (n = 16)	LP (n = 17)	OP (n = 15)
Age, y	27.11 ± 1.07	30.75 ± 0.82	24.88 ± 1.12*^a^*^,^*^d^*	29.33 ± 1.02
BMI, kg/m^2^	19.68 ± 0.34	29.00 ± 0.92*^b^*	19.63 ± 0.42*^d^*	31.56 ± 1.50*^b^*^,^*^d^*^,^*^f^*
FBG, mmol/L	4.65 ± 0.0.08	5.16 ± 0.24	4.47 ± 0.11	5.21 ± 0.29*^a^*^,^*^c^*^,^*^f^*
Fins, mIU/L	5.85 ± 0.33	14.04 ± 1.83*^a^*	10.83 ± 1.04*^a^*	36.98 ± 4.06*^b^*^,^*^d^*^,^*^f^*
HOMA-IR	1.21 ± 0.07	3.38 ± 0.54*^a^*	2.17 ± 0.24	9.15 ± 1.52*^b^*^,^*^d^*^,^*^f^*
TT, nmol/L	1.10 ± 0.09	1.23 ± 0.09	2.22 ± 0.20*^b^*^,^*^d^*	2.00 ± 0.19*^b^*^,^*^d^*
SHBG, nmol/L	85.17 ± 4.68	51.24 ± 6.10*^a^*	59.72 ± 10.48	16.66 ± 2.26*^b^*^,^*^d^*^,^*^f^*
DHEAS, *µ*g/mL	177.73 ± 12.43	177.30 ± 17.84	288.96 ± 33.85*^b^*^,^*^c^*	295.95 ± 23.64*^a^*^,^*^c^*
FAI	1.38 ± 0.14	2.53 ± 0.21	3.93 ± 0.42	15.39 ± 2.21*^b^*^,^*^d^*^,^*^f^*
TG, mmol/L	1.00 ± 0.21	2.11 ± 0.34*^b^*	1.98 ± 0.04*^a^*	2.67 ± 0.10*^b^*^,^*^e^*
TC, mmol/L	4.16 ± 0.25	4.51 ± 0.36	4.86 ± 0.22*^b^*^,^*^c^*	4.31 ± 0.20*^a^*^,^*^c^*^,^*^f^*
HDL, mmol/L	1.51 ± 0.07	1.22 ± 0.07*^a^*	1.56 ± 0.09*^c^*	1.04 ± 0. 06*^b^*^,^*^c^*^,^*^e^*
LDL, mmol/L	2.08 ± 0.10	2.93 ± 0.15*^a^*	2.47 ± 0.15	3.08 ± 0.19*^b^*^,^*^f^*

Data are mean ± standard error of the mean.

Abbreviations: FBG, fasting blood glucose; Fins, fasting blood insulin; TC, total cholesterol.

^*a*^ *P* < 0.05.

^*b*^ *P* < 0.01 compared with group LC.

^*c*^ *P* < 0.05.

^*d*^ *P* < 0.01 compared with group OB.

^*e*^ *P* < 0.05.

^*f*^ *P* < 0.01 compared with group LP.

### Serum profile of glycerophospholipid

Serum lipidomic changes associated with PCOS were analyzed by a shotgun lipidomic approach, including evaluation of phosphatidic acid, phosphatidylglycerol, phosphatidylserine (PS), phosphatidylinositol (PI), PC, and lysophospholipid of PC (LPC). This method routinely detects and quantifies ∼400 individual lipid ions, including PC, phosphatidylglycerol, PI, PS, phosphatidic acid, LPC, and ceramide. Among these lipids, we observed significantly higher levels of PC in the OP group than in the LC group, whereas, surprisingly, the concentrations of LPC were lower in the OP group than in the LC group (Table 2). However, there were no differences among the LP, LC, and OB groups. Further analyses of PC and LPC demonstrated that the PC species that were present at higher concentrations were C18:2, C20:2, C20:3, and C20:4, whereas the LPC species that were present at lower concentrations were C16:0, C18:0, and C18:1. Interestingly, we also found that the concentrations of ceramide in the OB, OP, and LP groups were significantly higher than those in the LC group.

**Table 2.** **Serum Phospholipids in Lean and Obese Patients and Patients With PCOS**

	LC	OB	LP	OP
PA, pmol/mL	2016.9 ± 345.6	3864.2 ± 1558.5	1938.3 ± 360.2	3314.9 ± 829.1
Phosphatidylglycerol, pmol/mL	1813.3 ± 445.9	3252.3 ± 856.5	390.7 ± 72.8*^d^*	2038.8 ± 615.1
PI, pmol/mL	3068.3 ± 492.8	4099.4 ± 780.1	1510.3 ± 276.9*^c^*	3399.3 ± 803.5
PS, pmol/mL	3211.2 ± 1519.9	1,3573.7 ± 6010.8	1183.3 ± 327.3	1,2974.2 ± 8466.0
Cer, pmol/mL	2375.8 ± 172.5	3154.1 ± 116.9*^a^*	3860.7 ± 297.6*^b^*^,^*^c^*	3105.2 ± 219.5*^a^*^,^*^e^*
LPC, nmol/mL	497.2 ± 66.0	355.9 ± 32.1	382.3 ± 80.7	314.2 ± 51.6*^a^*
PC, nmol/mL	619.3 ± 54.8	761.7 ± 44.9	792.0 ± 97.6	856.9 ± 59.2*^a^*
PC:D16:0–18:2	176.1 ± 17.3	203.9 ± 10.2	236.0 ± 39.4	249.9 ± 18.3*^a^*
PC:D18:2–18:2/D16:0–20:4	78.7 ± 8.6	107.1 ± 9.3	95.2 ± 23.3	107.2 ± 9.6
PC:D18:2–20:2/D18:0–20:4	50.4 ± 4.0	71.5 ± 6.6*^a^*	58.5 ± 8.0	66.6 ± 5.4*^a^*
PC:D18:1–18:2/D16:0–20:3	39.1 ± 5.2	44.7 ± 8.6	56.3 ± 6.9	58.2 ± 7.0*^a^*
PC:D18:0–18:2/D18:1–18:1	88.1 ± 9.2	98.2 ± 11.3	117.8 ± 11.6	127.7 ± 13.3*^a^*
LPC:16:0	133.2 ± 21.8	124.9 ± 21.7	76.3 ± 17.2*^a^*	85.7 ± 11.2*^a^*
LPC:P18:1	15.1 ± 4.8	4.8 ± 1.5*^a^*	10.8 ± 3.2	4.7 ± 0.98*^a^*
LPC: P18:0	19.2 ± 7.0	9.2 ± 2.6	12.5 ± 2.9	7.4 ± 2.4*^a^*
LPC:18:1	34.4 ± 5.7	24.6 ± 2.0	40.7 ± 11.2	20.2 ± 2.0*^a^*^,^*^e^*
LPC:18:0	53.8 ± 7.5	51.2 ± 7.1	48.8 ± 14.5	42.8 ± 8.1

Data are mean ± standard error of the mean.

Abbreviations: Cer, ceramide; PA, phosphatidic acid.

^*a*^ *P* < 0.05.

^*b*^ *P* < 0.01 compared with group LC.

^*c*^ *P* < 0.05.

^*d*^ *P* < 0.01 compared with group OB.

^*e*^ *P* < 0.05.

### Serum profile of FFAs

**Figure 2.**
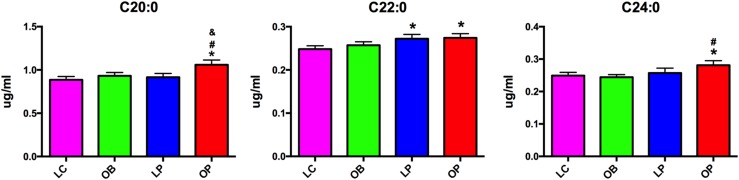
Serum saturated long-chain fatty acids in lean and obese patients and patients with PCOS. *P < 0.05 compared with LC; ^#^*P* < 0.05 compared with OB; ^&^*P* < 0.05 compared with LP.

Serum FFAs were analyzed using GC-MS. The concentrations of PUFAs, including LA (C18:2) and AA (C20:4), were significantly lower in the OP group than in the other 3 groups, whereas the concentration of DHA in the LP group was higher than in the LC and OP groups. By contrast, levels of the saturated long-chain fatty acids C20:0 and C24:0 were highest in the OP group, whereas the concentration of C22:0 was higher in the OP and LP groups than in the OB and LC groups (Fig. 2; Supplemental Table 1).

### Downstream metabolites of PUFAs

Accumulating evidence suggests that these lipid mediators play an important role in inflammation and obesity; therefore, the unique patterns of PUFA-containing PC and LPC that we observed in the OP group prompted us to further analyze the downstream bioactive lipids. There are 3 major enzymatic pathways that metabolize PUFAs. COXs convert AA into PGs, such as PGE_2_, PGD_2_, PGF_2_*_α_*, PGI_2_, and TXB_2_. Specific lipoxygenases (LOXs) metabolize AA into 5-, 12-, and 15-HETEs, and 5-LOX can also generate leukotrienes. Cytochrome P450 enzymes represent the third major metabolic pathway for AA, generating EETs, diHETEs, and 19-, or 20-HETEs. In addition to these 3 enzymatic pathways, free radical–induced lipid peroxidation generates structurally similar metabolites to the enzymatic products (34, 35). Some of these compounds, such as isoprostanes, can be used as markers for oxidative stress.

**Figure 3.**
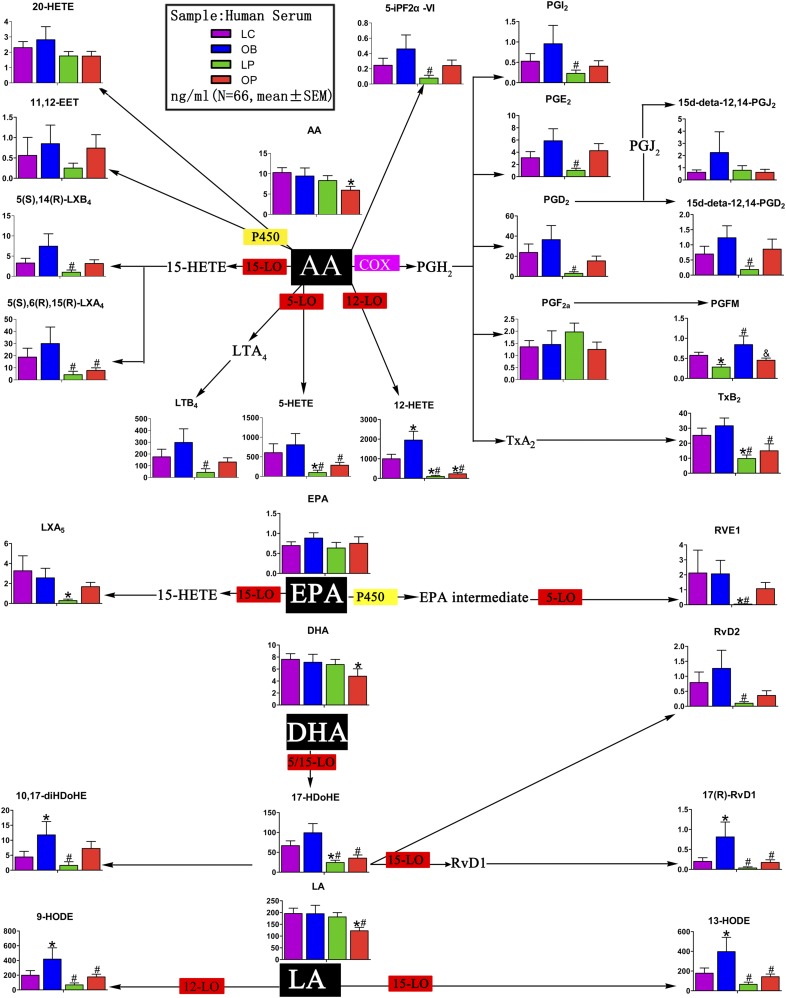
Serum bioactive lipids derived from AA, eicosapentaenoic acid (EPA), DHA, and LA. AA is C20:4; DHA is C22:6; EPA is C20:5; LA is C18:2. *P < 0.05 compared with LC; ^#^*P* < 0.05 compared with OB; ^&^*P* < 0.05 compared with LP. LT, leukotriene; LTB_4_, leukotriene B_4_; LX, lipoxin; LXA_4_, lipoxin A_4_; PGFM, prostaglandin F metabolite; PGI_2_, prostacycline I_2_; P450epo, cytochrome P450 epoxygenase; 15d-PGJ_2_, 15-deoxy-Δ^12,14^-prostaglandin J_2_.

Because of the low abundance of these downstream metabolites, we developed a targeted metabolomic approach to systematically quantify all of the major AA metabolites using liquid chromatography–MS and multiple reaction monitoring. As shown in Fig. 3 and Supplemental Table 2, the levels of COX metabolites, including PGI_2_, PGE_2_, PGD_2_, PG F metabolite, and TXB_2_, in the OB group, were higher than those in the LC group, whereas the levels of the same metabolites were decreased in the OP and LP groups vs control subjects. Similar patterns were also observed for the metabolites generated by the LOX and P450 pathways. Notably, the concentrations of leukotriene B_4_, 5-HETE, 12-HETE, 5(S),6(R),15(R)-LXA_4_, and 5(S),14(R)-LXB_4_ in the LP group were markedly lower than those in the OB group. Interestingly, metabolites of other PUFAs, such as LA, eicosapentaenoic acid, and DHA, showed a similar trend, *i.e.*, OB > LC > OP > LP (Fig. 3; Supplemental Table 2). Androgen attenuated the elevated production of bioactive lipid mediators that was associated with obesity. The differences in the metabolic profiles (LC compared with LP and OB compared with OP) remained after adjusting for age and BMI.

### Lipid profiles in obese rats and a rat model of PCOS

To further investigate the effects of obesity, hyperinsulinemia, and hyperandrogenism on the metabolism of AA and other PUFAs, we used a well-established high-fat-diet– and DHEA-induced rat model of obese PCOS (36). The model has a number of clinical and pathological features in common with obese patients with PCOS, such as obesity, hyperinsulinemia, hyperandrogenism, ovarian follicle immaturity, and ovulation disorders (37, 38). Compared with the control group (CON; normal diet), the HF group had significantly higher body mass and prominent hyperinsulinemia. Rats in the HF + DHEA group showed hyperandrogenism (Supplemental Table 3). Vaginal smears stained with Wright-Giemsa revealed that rats in the CON and HF groups had regular estrous cycles, whereas estrous cyclicity disappeared in the HF + DHEA group (Supplemental Fig. 1). Hematoxylin and eosin staining of ovarian specimens revealed that, unlike in the CON and HF groups, in HF + DHEA rats the number of corpora lutea was reduced dramatically, whereas the numbers of small and atretic follicles were increased. In addition, no mature or dominant follicles were observed (Supplemental Fig. 2). In summary, therefore, the HF + DHEA rat model showed insulin resistance, a lack of estrous cyclicity, hyperandrogenism, and follicular/ovulatory disorders, consistent with the main pathophysiological features of obese patients with PCOS.

Lipidomic studies were performed on rat serum and showed that total FFAs in the HF + DHEA group were significantly higher than in the CON and HF groups (Supplemental Table 4). In particular, AA was significantly higher in this group than in the other 2 groups (HF + DHEA, 22.24 ± 2.12 μg/mL, vs CON, 6.06 ± 0.57 μg/mL, and HF, 6.47 ± 0.35 μg/mL).

**Figure 4.**
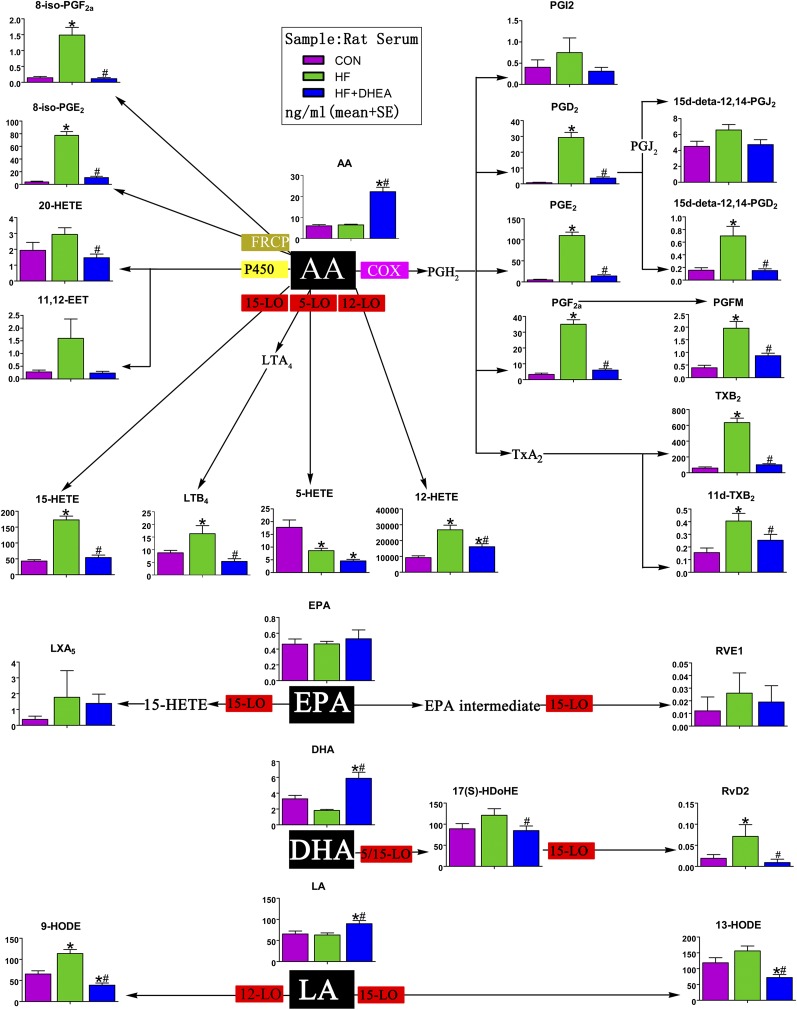
Lipid metabolites derived from AA, eicosapentaenoic acid (EPA), DHA, and LA in rat serum. AA is C20:4; DHA is C22:6; EPA is C20:5; LA is C18:2. *P < 0.05 compared with CON; ^#^*P* < 0.05 compared with HF. LTB_4_ leukotriene B_4_; LXA_4_, lipoxin A_4_; PGFM, prostaglandin F metabolite; PGI_2_, prostacycline I_2_; P450epo, cytochrome P450 epoxygenase; RVD, resolvin D; RVE, resolvin E; 15d-PGJ_2_, 15-deoxy-Δ^12,14^-prostaglandin J_2_.

Further analyses of AA metabolites generated by the 3 major enzymatic pathways (COX, LOX, and P450) revealed remarkably similar changes to those observed in the human clinical samples, *i.e.*, HF > CON > HF + DHEA (equivalent to OB > CON > OP). The changes in COX and LOX metabolites were significant. The differences in LA and DHA also resembled the pattern observed with the human clinical samples (Fig. 4; Supplemental Table 5).

## Discussion

PCOS is a complex and heterogeneous clinical syndrome. Women with this syndrome have an increased risk of developing T2DM and cardiovascular diseases. Emerging evidence suggests that chronic low-grade inflammation is involved in the pathogenesis of PCOS and obesity and is linked to insulin resistance (39). Besides the insulin resistance and associated compensatory hyperinsulinemia, hyperandrogenism is another core pathophysiological change of PCOS. However, the association between these pathological features and the characteristic changes in the lipid profiles of patients with PCOS has not been systematically investigated. This study provides an extensive profile of serum lipid metabolism in both humans and a rat model of PCOS and associated metabolic disorders, and clearly demonstrates that there are significant changes in the production of phospholipids, FFAs, and bioactive lipids in lean and obese patients with PCOS compared with women without PCOS.

Phospholipids are the main constituents of the cell membrane, and an altered profile may have a profound impact on cell function. Because of the complexity of phospholipid metabolism, lipidomics is the tool of choice to study systematic changes in phospholipids and to correlate these changes with disease. PC is 1 of the major phospholipids, and can be hydrolyzed to LPC and FFAs by phospholipases. The ratio of PC/LPC in serum has been studied in hepatic diseases associated with inflammation or infection. For example, LPC is decreased in a model of drug-induced liver injury, and in patients with viral hepatitis, alcoholic liver cirrhosis, and acute deterioration of liver function (40, 41). Furthermore, lower levels of LPC have been reported in patients with nonalcoholic steatohepatitis (42, 43). Thus, our observations of the features of phospholipid profiles in PCOS serum may reflect the inflammatory status of the liver, associated with the higher incidence of fatty liver in PCOS (Supplemental Fig. 3). The current study is consistent with a previous study in which serum LPCs (C16:0, C18:0, C18:1) were decreased in an animal model of fatty liver disease (44). AA in serum is primarily derived from the hydrolysis of phospholipids at sn-2 (45). Thus, the abnormal PC/LPC in the OP group may partially contribute to the decreased serum AA level.

Bioactive derivatives of AA, especially PGs, play a major role in inflammation and reproduction. Because serum AA was lower in the OP group, we also quantified the downstream metabolites of AA and other PUFAs using a targeted metabolomic approach. We found that similar patterns of metabolites were derived from the action of COX, LOX, and P450: their concentrations followed the pattern OB > LC > OP > LP. The most significant difference was between the OB and LP groups (Fig. 3; Supplemental Table 2). Interestingly, COX and LOX metabolites generated from other PUFAs, including eicosapentaenoic acid, DHA, and LA, showed patterns resembling those derived from AA (Fig. 3; Supplemental Table 2).

Whereas the OB group presented with obvious insulin resistance, but without marked hyperandrogenemia, the LP group had hyperandrogenemia and mild insulin resistance. Patients in the OP group were insulin resistant and hyperandrogenemic. On the basis of the differences in AA metabolites among the 4 groups, we hypothesize that insulin upregulates the expression and activity of COX, LOX, and other metabolic enzymes, whereas androgen downregulates their expression and/or activity. Because patients in the OP group had both insulin resistance and hyperandrogenemia, the levels of the metabolites in this group were in the midrange.

As shown in Table 1, fasting insulin was much higher in the OP group than in the other groups. Even in the presence of such a high concentration of insulin, however, the high androgen concentration still appeared to be capable of significantly lowering the production of bioactive lipids. These data, together with the fact that the levels of other metabolites in the OP group were close to, or even lower than, those in the control groups, strongly suggest that androgen can reverse or override the stimulatory effects of insulin. Alternatively, hyperinsulinemia may reduce the level of SHBG, leading to a high FAI value, and thereby amplification of the effect of androgens. The underlying molecular mechanisms of the antagonistic actions of insulin and androgen on bioactive lipids require further investigation.

Importantly, we successfully recapitulated data obtained from the human patients with PCOS in a well-established HF/DHEA-induced rat model of PCOS. Serum FFAs, including AA, were increased after injection of DHEA. The increase in free AA was most likely a result of the upregulation of lipolysis. In female animals, supraphysiological doses of androgens promote fat mobilization and decomposition (46, 47). Our polymerase chain reaction results demonstrated that the expression of hormone-sensitive lipase messenger RNA was significantly upregulated in the adipose tissue of the HF + DHEA group vs the CON and HF groups (Supplemental Fig. 4).

In the HF group, although there was no significant change in AA compared with controls, AA metabolites were markedly increased, which is consistent with the clinical findings (Fig. 4). Even with the significant increase of AA in the HF + DHEA group, we observed a profound decrease in the main AA metabolites, which suggests a strong inhibitory effect of androgen on bioactive lipid production. Similar results were also obtained with regard to LA, DHA, and other PUFA metabolites (Fig. 4; Supplemental Table 5). The data obtained from the animal studies were consistent with those from the human studies: obesity, which is commonly accompanied by compensatory hyperinsulinemia, promotes PUFA metabolism, whereas androgen has an inhibitory effect on PUFA metabolism.

It is well established that obesity is associated with chronic low-grade inflammation. Previously, increased levels of circulating androgens in obese women with PCOS had been thought to lead to enhanced inflammation, which would aggravate the metabolic disorder. However, recent studies show that the elevated circulating androgens may exert anti-inflammatory effects when obesity is present (48, 49). Nonetheless, the underlying molecular mechanism remains to be elucidated. Our data show that the COX, LOX, and P450 metabolic pathways were upregulated in obese women, which resulted in higher levels of bioactive lipids. Conversely, concentrations of these bioactive lipids were lower when hyperandrogenism was present, as in both the obese patients with PCOS and the obese PCOS rat model. Thus, our data provide insights into an anti-inflammatory role of androgens, which is at least partially mediated by a reduction in AA-derived inflammatory lipids.

Androgens also play an important part in lipid metabolism. Previous studies have focused mainly on cholesterol (total, HDL, and LDL), oxidized LDL, and TGs and have shown that androgens also increase lipolysis in visceral fat cells in women with PCOS (15, 16). To our knowledge, however, the effects of androgen on the metabolic profiles of PUFA, especially in obese women and women with PCOS, have not been reported. Our observations that androgens antagonize the production of PGs may have clinical implications for the reproductive disorders present in PCOS.

COX2 is involved in various reproductive functions in the ovary, including oocyte maturation, ovulation, early embryonic development, implantation, and parturition, as previously mentioned (16–18). Nevertheless, the effects of androgen on COX2 expression and activity remain controversial. Yazawa *et al.* (50) found that dihydrotestosterone (DHT) treatment induced expression of the COX2 gene in granulosa cells from normal rats and human ovarian granulosa-like tumor cell line (KGN) *in vitro*, whereas other studies suggest a more complicated method of COX2 regulation by DHT. For example, although DHT upregulated COX2 expression in the absence of induced inflammation *via* an androgen receptor (AR)–dependent mechanism, it downregulated COX2 expression in cytokine- or LPS-induced inflammation *via* an AR-independent mechanism (51, 52). Thus, DHT differentially influences COX2 levels through AR-dependent and independent mechanisms, depending on the physiological and pathophysiological state of the cells (51). If the follicle is in an inflammatory microenvironment, COX2 expression may be inhibited by the elevated androgen, and the luteinizing hormone–induced PG levels will be decreased, eventually resulting in impaired spontaneous ovulation in women with PCOS. We are currently investigating the molecular mechanisms whereby hyperandrogenism contributes to ovulatory dysfunction through modulation of bioactive lipids, including PGs.

In summary, we observed an abnormal PC/LPC ratio in obese patients with PCOS, which may result in changes in serum AA levels. Obesity, which is usually accompanied by compensatory hyperinsulinemia, promoted the metabolism of AA and other PUFAs, whereas androgens had an inhibitory effect. Further understanding of the molecular mechanisms that lead to the altered lipid profiles we identified, together with genomic and proteomic studies, may provide new insights into the pathogenic mechanisms of PCOS and suggest novel potential therapeutic strategies.

## Abbreviations:

AA: arachidonic acid
AR: androgen receptor
BMI: body mass index
CON: control group
COX: cyclooxygenase
DHA: docosahexaenoic acid
DHEAS: dehydroepiandrosterone sulfate
DHT: dihydrotestosterone
EET: epoxyeicosatrienoic acid
FAI: free androgen index
FFA: free fatty acid
GC: gas chromatography
HDL: high-density lipoprotein
HETE: hydroxyeicosatetraenoic acid
HF: high-fat-diet–fed group
HF + DHEA: high-fat-diet–fed and hyperandrogenism group
HODE: hydroxyoctadecadienoic acid
HOMA-IR: homeostatic model assessment–insulin resistance
LA: linoleic acid
LC: lean control
LDL: low-density lipoprotein
LOX: lipoxygenase
LP: lean with polycystic ovary syndrome
LPC: lysophospholipid of phosphatidylcholine
MS: mass spectrometry
OB: obese
OP: obese with polycystic ovary syndrome
PC: phosphatidylcholine
PCOS: polycystic ovary syndrome
PG: prostaglandin
PI: phosphatidylinositol
PS: phosphatidylserine
PUFA: polyunsaturated fatty acid
SHBG: sex hormone–binding globulin
TG: triglyceride
TT: total testosterone
TXB2: thromboxane B2
T2DM: type 2 diabetes mellitus
